# Development of Novel Analogs of the Monocarboxylate Transporter Ligand FACH and Biological Validation of One Potential Radiotracer for Positron Emission Tomography (PET) Imaging

**DOI:** 10.3390/molecules25102309

**Published:** 2020-05-14

**Authors:** Masoud Sadeghzadeh, Barbara Wenzel, Daniel Gündel, Winnie Deuther-Conrad, Magali Toussaint, Rareş-Petru Moldovan, Steffen Fischer, Friedrich-Alexander Ludwig, Rodrigo Teodoro, Shirisha Jonnalagadda, Sravan K. Jonnalagadda, Gerrit Schüürmann, Venkatram R. Mereddy, Lester R. Drewes, Peter Brust

**Affiliations:** 1 Department of Neuroradiopharmaceuticals, Institute of Radiopharmaceutical Cancer Research, Helmholtz-Zentrum Dresden-Rossendorf, Permoserstraße 15, 04318 Leipzig, Germany; b.wenzel@hzdr.de (B.W.); d.guendel@hzdr.de (D.G.); w.deuther-conrad@hzdr.de (W.D.-C.); m.toussaint@hzdr.de (M.T.); r.moldovan@hzdr.de (R.-P.M.); s.fischer@hzdr.de (S.F.); f.ludwig@hzdr.de (F.-A.L.); r.teodoro@hzdr.de (R.T.); p.brust@hzdr.de (P.B.); 2 Department of Chemistry and Biochemistry, Department of Pharmacy Practice & Pharmaceutical Sciences, University of Minnesota, Duluth, MN 55812, USA; sgurrapu@d.umn.edu (S.J.); skjonnal@d.umn.edu (S.K.J.); vmereddy@d.umn.edu (V.R.M.); 3 UFZ Department of Ecological Chemistry, Helmholtz Centre for Environmental Research, Permoserstraße 15, 04318 Leipzig, Germany; gerrit.schuurmann@ufz.de; 4 Institute of Organic Chemistry, Technical University Bergakademie Freiberg, Leipziger Straße 29, 09599 Freiberg, Germany; 5 Department of Biomedical Sciences, University of Minnesota Medical School Duluth, 251 SMed, 1035 University Drive, Duluth, MN 55812, USA; ldrewes@d.umn.edu

**Keywords:** monocarboxylate transporters (MCTs), FACH, ^18^F-labeled analog of FACH, α-CCA, blood-brain barrier (BBB), positron emission tomography (PET) imaging

## Abstract

Monocarboxylate transporters 1-4 (MCT1-4) are involved in several metabolism-related diseases, especially cancer, providing the chance to be considered as relevant targets for diagnosis and therapy. [^18^F]FACH was recently developed and showed very promising preclinical results as a potential positron emission tomography (PET) radiotracer for imaging of MCTs. Given that [^18^F]FACH did not show high blood-brain barrier permeability, the current work is aimed to investigate whether more lipophilic analogs of FACH could improve brain uptake for imaging of gliomas, while retaining binding to MCTs. The 2-fluoropyridinyl-substituted analogs **1** and **2** were synthesized and their MCT1 inhibition was estimated by [^14^C]lactate uptake assay on rat brain endothelial-4 (RBE4) cells. While compounds **1** and **2** showed lower MCT1 inhibitory potencies than FACH (IC_50_ = 11 nM) by factors of 11 and 25, respectively, **1** (IC_50_ = 118 nM) could still be a suitable PET candidate. Therefore, **1** was selected for radiosynthesis of [^18^F]**1** and subsequent biological evaluation for imaging of the MCT expression in mouse brain. Regarding lipophilicity, the experimental log D_7.4_ result for [^18^F]**1** agrees pretty well with its predicted value. In vivo and in vitro studies revealed high uptake of the new radiotracer in kidney and other peripheral MCT-expressing organs together with significant reduction by using specific MCT1 inhibitor α-cyano-4-hydroxycinnamic acid. Despite a higher lipophilicity of [^18^F]**1** compared to [^18^F]FACH, the in vivo brain uptake of [^18^F]**1** was in a similar range, which is reflected by calculated BBB permeabilities as well through similar transport rates by MCTs on RBE4 cells. Further investigation is needed to clarify the MCT-mediated transport mechanism of these radiotracers in brain.

## 1. Introduction

Monocarboxylate transporters (MCTs), comprising 14 isoforms, are dedicated to the solute carrier 16 (*SLC16*) gene family [1,2]. Of all the MCT isoforms, MCT1-4 are well characterized and known as membrane-bound carriers that bidirectionally transport short-chain monocarboxylic acids, most notably L-lactate, pyruvate, and ketone bodies along with protons across the plasma membrane of mammalian cells [2]. The tissue distribution of the MCT isoforms is quite variable. Although MCT1 is ubiquitously distributed in the muscles, it is additionally expressed along with MCT4 in the brain and other peripheral organs like small intestine, liver, heart, kidney, and blood cells [1,3]. Aberrant expression such as, upregulation of MCT1 and MCT4 has been reported in a large number of tumors (e.g., neuroblastomas, high-grade gliomas, carcinomas of renal cells, breast epithelium, colorectal and squamous tissues, and cervical and lung cancers) where expression is correlated to poor outcomes. In these tissues, the MCTs serve to facilitate the shuttling of lactate between cells with different metabolic requirements [4,5,6]. Due to the metabolic reprogramming, considered as a hallmark of cancer, tumor cells indeed switch from glucose to lactate as a crucial energy supply, hence, their metabolism heavily relies on glycolysis and consequently the lactate efflux through MCT1 and MCT4 in order to prevent their own acidosis and to regenerate NAD^+^ [7]. Accordingly, both transporters are attractive therapeutic and even diagnostic targets for the treatment and detection of human cancers [4,7,8].

Positron emission tomography (PET) is known as a powerful tool for non-invasive molecular detection of early metabolic changes in cancer progression [9]. [^18^F]Fluorodeoxyglucose ([^18^F]FDG), a radiolabeled glucose analog, is well known as a standard PET tracer used for diagnosis, staging and treatment monitoring in clinical oncology [10,11]. Considering the lack of specificity and sensitivity of [^18^F]FDG for several types of tumors [10], there is still an unmet clinical need for cancer detection and therapy. Thus, the complementary concept based on components of the aerobic glycolysis and metabolism by malignant cells is more intriguing and potentially rewarding [2,12,13,14]. In this regard, [^18^F]DASA-23, recently developed as a potent radiotracer for imaging tumor glycolysis by targeting pyruvate kinase M2, is currently in phase I clinical trials (ClinicalTrials.gov, NCT03539731) [15]. Because the metabolic reprogramming in cancer cells may also result in the overexpression of MCT1/MCT4 in many cancers [4,7], MCT-targeting PET studies provide an opportunity to achieve more accurate and useful understanding of certain aspects of the tumor-specific metabolism [8,16]. 

**Figure 1:**
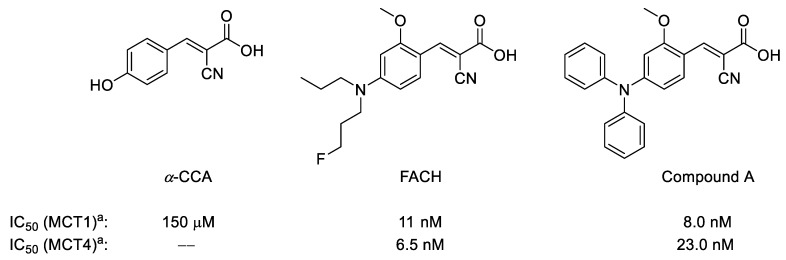
Chemical structures of α-CCA and its novel derivatives as potent monocarboxylate transporters 1/4 (MCT1/MCT4) inhibitors; ^a^ Inhibition of [^14^C]lactate uptake was determined in RBE4 cells (for MCT1) and in MDA-MB-231 cells (for MCT4) by measuring intracellular radioactivity after 60 min incubation without and with the respective inhibitor at 37 °C [25].

During the last decade, only a few ^11^C- or ^18^F-labeled substrates of MCTs such as [^11^C]lactate, [^11^C]pyruvate as well as their ^18^F-labeled analogs were investigated for imaging of MCTs by PET [17,18,19,20]. To the best of our knowledge, only limited examples of the MCTs inhibitors were investigated as PET radiotracers for in vivo applications [21]. Although one of the best characterized inhibitors, α-cyano-4-hydroxycinnamic acid (α-CCA), possesses a 10-fold selectivity for MCT1 compared to other subtypes (Figure 1) [22], it also shows significant inhibitory potency towards the mitochondrial pyruvate carrier in isolated mitochondria [23]. Accordingly, very potent and more specific MCT1/MCT4 inhibitors have been developed based on a comprehensive structure-activity relationship study on a series of α-CCA derivatives [24,25]. Based on this approach, we recently developed and evaluated [^18^F]FACH as the first ^18^F-labeled inhibitor of MCTs (Figure 1) [26]. Along with a high inhibitory potency towards MCT1 (11.0 nM) and MCT4 (6.5 nM) [26], [^18^F]FACH showed very promising pharmacokinetics in healthy mice, in particular in the kidneys, as organ with a high physiological expression of MCT1 [1,3,27]. 

Nonetheless, [^18^F]FACH showed only moderate brain uptake, which might be related to the rather hydrophilic features (log D_7.4_ = 0.42) [28], assuming that [^18^F]FACH could only passively enter the brain. This assumption would be in accordance with the fact that for the structural analog α-CCA, the site of its MCT inhibition has been demonstrated to be the extracellular surface [29,30]. Moreover, it is well established that MCT1 is also the prominent monocarboxylic acid transporter in the cerebral microvascular endothelium, facilitating the bidirectional transport of lactate through brain endothelial cells and the blood-brain barrier (BBB) [31]. Many brain tumors, such as gliomas and neuroblastomas, produce high amounts of lactic acid and consequently up-regulate MCT1, thus, inducing acidosis in the tumor microenvironment [5]. MCT1 is therefore proposed as a most likely therapeutic target for neuroblastomas and gliomas, and α-CCA has been able to suppress tumor growth via inhibition of MCT1 [5,32,33]. Accordingly, the development of MCT1-targeting radiotracers possessing sufficiently high brain permeability would be an important step forward toward brain imaging. 

Although α-CCA as well as its new analog FACH (Figure 1) contain a Michael acceptor unit, their highly predominating carboxylate form under physiological conditions (ACD-calculated p*K*_a_ < 1 vs. pH 7.4; see Table 1 below) masks a respective electrophilic reactivity. This implies that these compounds are most likely not active as protein-attacking electrophiles. Moreover and as mentioned above, α-CCA has been reported to remain extracellular, inhibiting MCT from the outside in a competitive manner and without adverse effects under therapeutic concentrations [29,30].

**Figure 2:**
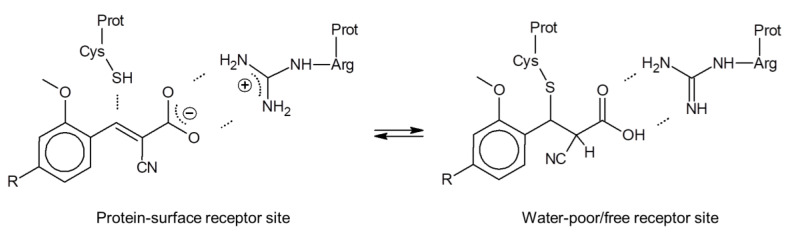
Hypothetical MCT-inhibitor binding at the extracellular surface (left) and at an interior protein site with no aqueous solvation (right). Michael addition may become active for the neutral carboxylic acid form (right), but would be reversible due to the α-CN substitution that enhances the retro-Michael reaction significantly (see, e.g., [34,35]).

In this context, it is interesting that for all respective cinnamic acid derivatives, the electron-withdrawing α-CN substituent is required for their MCT inhibition potency, which holds also for FACH [26]. As a possible explanation, we hypothesize that their non-covalent interaction with the MCT protein at the cellular surface may include an electrostatic Arg-carboxylate binding motif as primary anchor. This may facilitate a further complex stabilization through approaching a Cys-thiol by the Michael acceptor β-carbon that is activated further through the α-CN substitution (Figure 2, left). To balance the carboxylate anionic charge, a separate extracellular proton, required for the MCT action as respective symporter, could be attached temporarily at a His-nitrogen (not shown in Figure 2). In case of a successful carboxylate transport such as for the lactate efflux, a respective (additional) His-proton could be liberated to the interstitial compartment.

In case the Arg-carboxylate interaction would result in a charge compensation sufficient to unmask the Michael acceptor reactivity, the Cys-thiol might add to the α,β-unsaturated unit, possibly following a proton transfer from Arg to the carboxylate (right part of Figure 2). In this case, however, the α-CN substituent enhances both the Michael and the retro-Michael reactivity, making this covalent reaction reversible through stabilization of the carbanion intermediate [34,35]. In conclusion, we hypothesize that despite the Michael acceptor unit common to all cinnamic acid derivatives, their mode of action is probably non-covalent or under water-poor/free conditions at least only temporarily covalent, with a correspondingly negligible risk to form permanent covalent bonds to nucleophilic protein sites. Results from a respective toxicity study will be reported in due course. 

**Figure 3:**
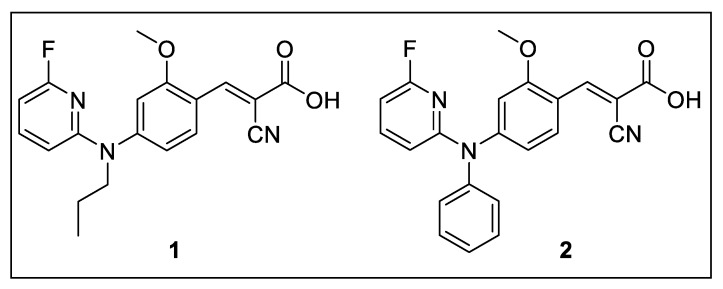
New analogs of FACH investigated in the current study.

With the goal to improve the brain uptake by passive diffusion, we designed new analogs of FACH by replacing the less lipophilic propyl groups with more lipophilic aryl and heteroaryl moieties (Figure 3). Notably, the structurally modified analogs yet need to retain an acceptable inhibitory potency towards MCT1. On the basis of compound A, which was reported to exhibit high MCT1 inhibition (IC_50_ = 8.0 nM, Figure 1) [25], two fluorinated analogs were developed by introducing 2-fluoropyridinyl and phenyl groups (compounds **1** and **2**, Figure 3). Herein we describe the organic synthesis of the new compounds and their inhibitory potency for MCT1-mediated lactate transport. Furthermore, radiofluorination of **1** was performed and the resulting new radiotracer [^18^F]**1** was investigated in mice to assess the impact of higher lipophilicity on the in vivo features compared to [^18^F]FACH for imaging of MCT1 in mouse brain. 

## 2. Results and Discussion

### 2.1. Organic Chemistry and Monocarboxylate Transporter Inhibition

**Scheme 1:**
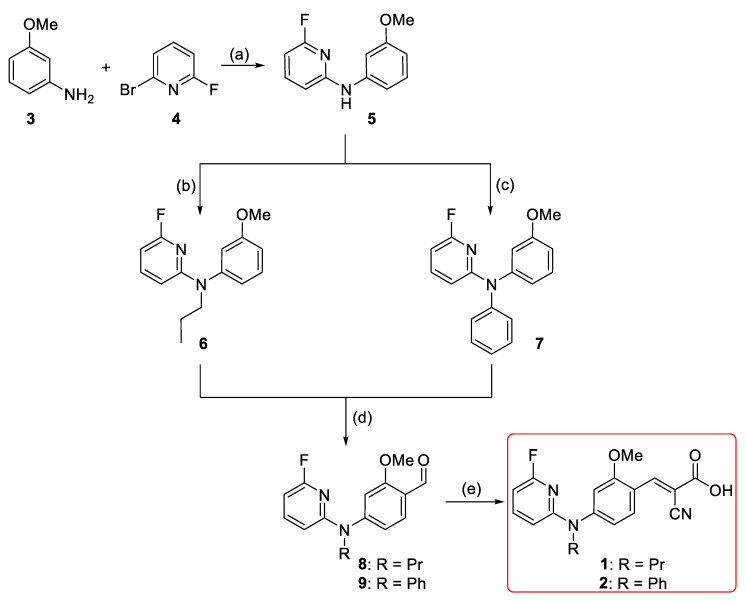
Synthesis of **1** and **2**; reagents and reaction conditions: (a) Pd(OAc)_2_ (5 mol %), Xantphos (5 mol %), Cs_2_CO_3_, 1,4-dioxane, Ar, 105 °C, 50 min, 96%; (b) 1-iodopropane, NaH (60% oil dispersion), DMF, Ar, r.t., 1.5 h, 95%; (c) PhBr, Pd(OAc)_2_ (15 mol %), Xantphos (15 mol %), Cs_2_CO_3_, 1,4-dioxane, Ar, 105 °C, 24 h, 46%; (d) POCl_3_, DMF, Ar, 80 °C, 2–4 h, 57% (for **8**) and 68% (for **9**); (e) *i.* cyanoacetic acid, piperidine, ACN, reflux; *ii.* HCl (6 M), r.t. 30 min, 95% (for **1**) and 98% (for **2**).

For developing the compounds **1** and **2,** the di-arylamine intermediate **5** was synthesized via the Buchwald-Hartwig aryl amination according to the previously reported procedures (Scheme 1) [36,37]. Alkylation of 6-fluoro-*N*-(3-methoxyphenyl)pyridin-2-amine **5** using 1-iodopropane and sodium hydride afforded **6** in 95% yield [38]. Compound **7** was obtained via a second Buchwald–Hartwig amination of **5** with phenyl bromide in negligible yield (< 10%). However, a stepwise addition of the palladium (Pd) catalyst and the phosphine ligand together with a longer reaction time led to the formation of **7** in moderate yield (46%). This might be related to the decreased electron density of the nitrogen atom due to the 2-fluoropyridinyl substituent and/or the steric hindrance effect. Both **6** and **7** were afterwards subjected to Vilsmeier–Haack formylation [39] to afford **8** and **9** with yields of 57% and 68%, respectively. Finally, Knoevenagel condensation of aldehydes **8** and **9** with cyanoacetic acid generated **1** and **2** in nearly quantitative yields (Scheme 1) [26].

Inhibition of MCT1-mediated lactate transport of **1** and **2** was investigated by [^14^C]lactate uptake assays using immortalized rat brain endothelial-4 cells (RBE4) [40] which express mainly MCT1 [24,25]. Both compounds dose-dependently inhibited the lactate uptake, with IC_50_ values of 118 nM (**1**) and 274 nM (**2**). Accordingly, replacing the 1-fluoropropyl group of FACH by a 2-fluoropyridinyl group in **1** resulted in a 10-fold decrease of the inhibitory potency. When comparing **2** with compound A, the substitution of the phenyl ring by a 2-fluoropyridinyl ring in **2** (Figure 1) caused an even stronger reduction of the inhibitory potency [25]. We therefore decided to proceed with **1** for radiofluorination and biological evaluation.

**Scheme 2:**
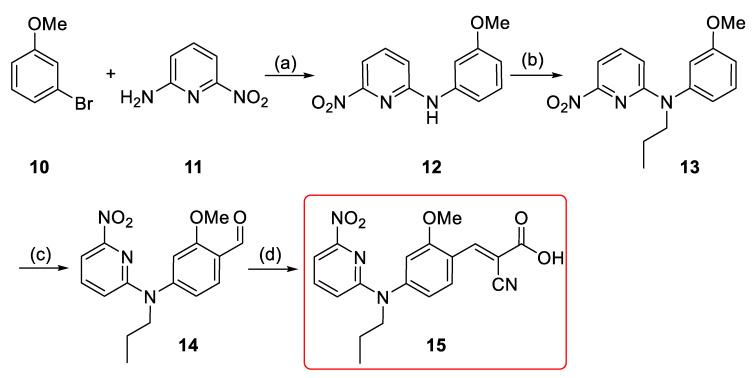
Synthesis of the nitro precursor (**15**): Reagents and reaction conditions: (a) Pd(OAc)_2_ (5 mol %), Xantphos (5 mol %), Cs_2_CO_3_, 1,4-dioxane, Ar, 105 °C, 2 h, 80%; (b) 1-iodopropane, NaH (60% oil dispersion), DMF, Ar, r.t., 1.5 h, 93%; (c) POCl_3_, DMF, Ar, 80 °C, 1.5 h, 92%; (d) i. cyanoacetic acid, piperidine, ACN, reflux, 5 h; ii. HCl (6 M), r.t. 30 min, above 98%.

In order to develop the new MCT1-targeting radiotracer [^18^F]**1**, a precursor including a suitable leaving group was required for the nucleophilic aromatic substitution (S_N_Ar) with [^18^F]fluoride. A nitro precursor (**15**) with an unprotected carboxylic acid function (Scheme 2) was synthesized considering the good results obtained for the aliphatic nucleophilic substitution with unprotected precursor in the one-step radiosynthesis of [^18^F]FACH [28]. Initial Buchwald–Hartwig aryl amination between 2-amino-6-nitropyridine and 3-bromoanisole provided the *N*-substituted anisidine **12** in 80% yield (Scheme 2) [36]. Alkylation of **12** under basic condition provided **13** with a yield of 93% [38]. Vilsmeier–Haack formylation of **13** gave aldehyde **14** in 92% yield [39]. It was followed by Knoevenagel condensation with cyanoacetic acid to provide **15** in 67% overall yield [26]. The chemical purity of the precursor **15** was > 98%, according to NMR and HPLC analyses. 

### 2.2. Radiosynthesis, Stability, and Determination of log D_7.4_

**Scheme 3:**
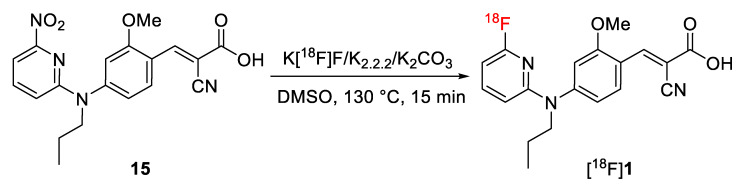
Radiosynthesis of [^18^F]**1**.

As shown in Scheme 3, [^18^F]**1** was synthesized on the basis of an S_N_Ar reaction via substitution of the NO_2_ leaving group of **15** by [^18^F]fluoride in the presence of Kryptofix^®^ (K_2.2.2_) and K_2_CO_3_. In dimethylsulfoxide (DMSO), the S_N_Ar reaction proceeded smoothly and resulted in high radiochemical yields of 73 ± 12% (n = 4, non-isolated, radio-HPLC) for [^18^F]**1** after 15 min conventional heating at 130 °C. Besides unreacted [^18^F]F^−^, radioactive by-products accounted for less than 5%. [^18^F]**1** was isolated by semi-preparative HPLC (Supplementary Data, Figure S1A), trapped on a pre-conditioned Sep-Pak C18 light cartridge, eluted with ethanol, and formulated in isotonic saline containing 10% of EtOH (*v*/*v*) for better solubility. Analytical radio- and UV-HPLC analyses of the final product co-eluted with the reference **1**, confirmed the identity of the radiotracer (Supplementary Data, Figure S1B). Finally, [^18^F]**1** was obtained with radiochemical yields of 51 ± 11% (n = 3, decay-corrected to the end of the bombardment) in a total radiosynthesis time of about 90 min, at a radiochemical purity of ≥ 98% and with molar activities in the range of 180–200 GBq/µmol (n = 3, end of synthesis) using starting activities of 2–3 GBq.

The stability of the radiotracer was investigated by incubation of [^18^F]**1** in *n*-octanol, saline, phosphate-buffered saline (PBS) and ethanol. Samples were analyzed by radio-thin-layer chromatography (TLC) and radio-HPLC and no degradation or defluorination was observed in any of the solvents after 60 min incubation at 40 °C. 

A variety of physicochemical parameters affects the brain permeability of different brain-targeting radiotracers [41]. Lipophilicity, often, but not necessarily, correlates with the ability to cross the BBB, and is considered as an important physicochemical property. In Table 1, calculated bioavailability-related parameters are listed for α-CCA, FACH and selected structural analogs. Accordingly, the new derivatives **1** and **2** show the desired higher hydrophobicity (log *K*_ow_ = logarithmic *n*-octanol/water partition coefficient [42], log D_7.4_ = log *K*_ow_ corrected for ionization at pH 7.4 [43,44]) as compared to FACH. Nevertheless, Table 1 also shows that the predicted brain-blood partition coefficients (log *K*_BB_ [43]) of **1** (–0.49) and **2** (–1.05) are below the one of FACH (–0.10).

Note further that FACH is both more lipophilic and more BBB-permeable than α-CCA. Regarding the Michael-acceptor unit mentioned above, comparison of cinnamic acid and its α-CN derivative shows that the α-CN substitution decreases the p*K*_a_ value by 3.7 units, most likely because of its combined inductive and mesomeric electron-withdrawing effect. Accordingly, all α-CCA derivatives are significantly acidic with p*K*_a_ values below 1, indicating for all of them that the dissociated carboxylate form is prevalent under physiological conditions. Experimental investigation of the lipophilicity of [^18^F]**1** through employing the shake-flask method using *n*-octanol and PBS (pH 7.4) resulted in a log D_7.4_ value of 0.820 ± 0.003 (n = 4). This value agrees pretty well with its calculated counterpart of 1.08 (Table 1), which holds correspondingly for the FACH log D_7.4_ value (0.42 experimental [28] vs. 0.69 calculated) as well as for the respective difference in log D_7.4_ values.

### 2.3. In Vitro and In Vivo Biological Validation of [^18^F]**1**

**Figure 4:**
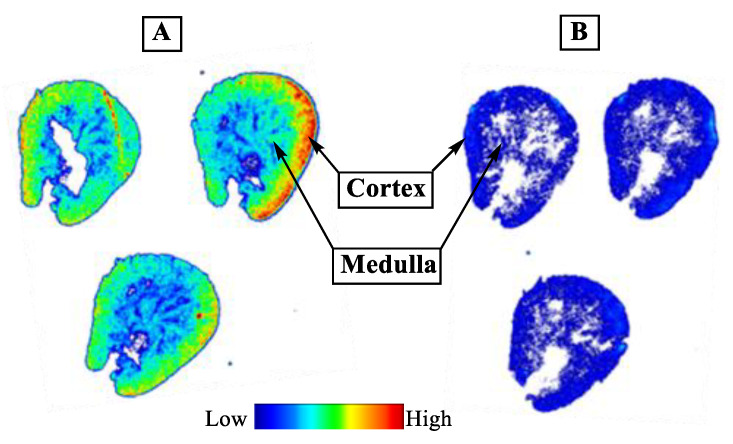
In vitro autoradiography of [^18^F]**1** in transversal cryosections of the mouse kidney. Total (**A**) and non-specific (**B**) binding of ~1 nM [^18^F]**1** obtained without and with co-incubation with 10 µM α-CCA-Na.

It is well demonstrated that several members of the MCT family are highly expressed in mammalian kidney, where over 95% of the lactate reabsorption takes place [1,3,45,46]. MCT1 mRNA and protein have clearly been detected on both the human kidney derived cell line HK-2 and human kidney cortex. In HK-2 cells it was found exclusively on the basal membrane [45]. Therefore, the specific binding of [^18^F]**1** to MCTs was initially proven by in vitro autoradiography using cryosections of the mouse kidney. As reflected by the autoradiographic images presented in Figure 4, co-incubation of ~1 nM [^18^F]**1** with 10 µM α-CCA-Na resulted in significantly lower binding of the radiotracer. Therefore, the binding of [^18^F]**1** in mouse kidney in vitro is highly specific.

**Figure 5:**
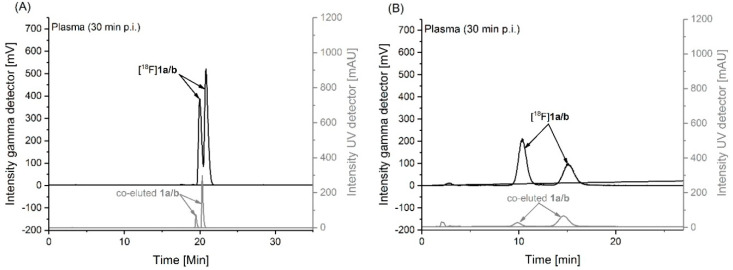
Analytical UV- and radio-HPLC chromatograms representing two peaks a/b which are supposed to reflect the neutral and deprotonated form of the radiotracer ([^18^F]**1a/b**) in mouse plasma at 30 min p.i. measured under: (**A**) reversed phase (Reprosil-Pur C18-AQ, 250 × 4.6 mm, gradient with an eluent mixture of ACN/20 mM NH_4_OAc (aq.), 370 nm, 1.0 mL/min), and (**B**) micellar conditions (Reprosil-Pur C18-AQ, 250 × 4.6 mm, isocratic mode with water containing 50 mM sodium dodecyl sulfate/10 mM NaHPO_4_, 1.0 mL/min).

To investigate the stability of [^18^F]**1** in vivo, samples of plasma and brain homogenates obtained from CD-1 mice at 30 min after intravenous injection of the radiotracer were analyzed for radiometabolites by using reversed-phase and micellar (MLC) radio-HPLC. MLC allows a direct injection of the samples into the HPLC system without the elimination of the tissue matrix as already described [47,48]. In general, the results obtained with both methods are comparable and the analyses revealed solely intact radiotracer and no detectable radiometabolites in plasma (Figure 5A–B) and brain (Supplementary Data, Figure S2) samples. Notably, in both samples two peaks a/b were detected by analytical HPLC (Figure 5/Figure S2) which are supposed to represent the neutral and deprotonated form of the radiotracer ([^18^F]**1a/b**). This finding suggests that the analytical HPLC conditions do not reflect the physiological milieu at which the neutral compound fraction would be negligible according to the Henderson–Hasselbalch equation. Research into the speciation of **1** under analytical-chemical conditions is subject to a future investigation.

**Figure 6:**
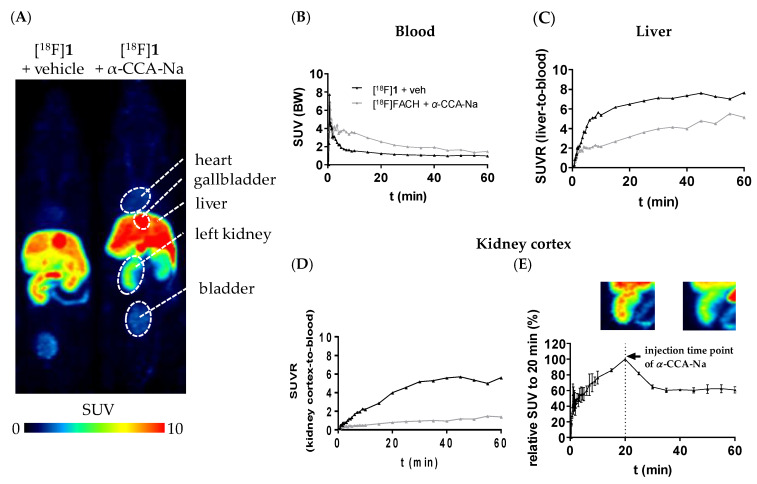
(**A**) Maximum intensity projections (MIPs) of small animal positron emission tomography (PET) images (45–60 min p.i., n = 1) of female CD-1 mice depicting the differential distribution of [^18^F]**1** in peripheral organs without and with pre-administration of α-CCA-Na; (**B**–**D**) Time-activity curves representing the SUV of blood, as well as the standardized uptake value (SUV) ratio (SUVR) from liver-to-blood and kidney cortex-to-blood; (**E**) displacement study: i.v. application of α-CCA-Na 20 min after tracer injection (n = 2), MIPs of the left kidney (left: 15–20 min p.i. and right: 45–60 min p.i.).

Pharmacokinetic studies of [^18^F]**1** were performed by dynamic PET imaging in mouse using a dedicated small animal PET/MR camera. The target-specificity of [^18^F]**1** was investigated by pre-administration of the blocking compound α-CCA-Na. Maximum intensity projections of PET studies from a representative control and α-CCA-Na treated animals and time-activity curves (TACs) from tissues of interest are presented in Figure 6. [^18^F]**1** cleared rapidly from the blood with an initial TAC peak standardized uptake value (SUV) of 7.3 and a SUV of 1.5 after 10 min followed by a slow blood clearance to a SUV of 0.9 after 60 min in the control group (Figure 6B). Pre-administration of the MCT inhibitor α-CCA-Na resulted in an initial TAC peak SUV of 6.9 which was comparable to the control group, whereas a higher SUV of 3.5 after 10 min and a SUV of 1.5 was reached after 60 min p.i., reflecting higher availability of the radiotracer in the blood (Figure 6B). This is expected to be caused by blocking the uptake of [^18^F]**1** in peripheral organs in vivo. In comparison to the control conditions, the pre-administration of α-CCA-Na significantly reduced the activity accumulation in the MCT1-expressing renal cortex [46] throughout the whole imaging period, which is shown by the SUV ratio (SUVR) of kidney cortex-to-blood (Figure 6D). Furthermore, the displacement study revealed 39.2% drop of the SUV, 20 min after i.v. injection of α-CCA-Na (Figure 6E), which implicates a reversible tissue uptake of [^18^F]**1** in the kidney cortex. Nevertheless, further studies are needed to clarify the exact mechanism of the radiotracer uptake. Regarding liver, where the highly expressed MCT1 transports L-lactate into the parenchymal cells for gluconeogenesis [1], a constantly increasing accumulation of activity can be observed under both control and blocking conditions, although at lower values under pre-administration of α-CCA-Na (Figure 6C). 

Taking into consideration the high activity concentrations persistently accumulated in the kidney and liver, the blocking effect of α-CCA-Na in both tissues will result in a strong increase in the fraction of available tracer in blood as reflected by the higher SUV in blood observed in the blocking experiments (Area Under the Curve (AUC)_0–60 min_ = 140 SUV × minutes) compared to the control experiments (AUC_0–60 min_ = 75 SUV × minutes).

**Figure 7:**
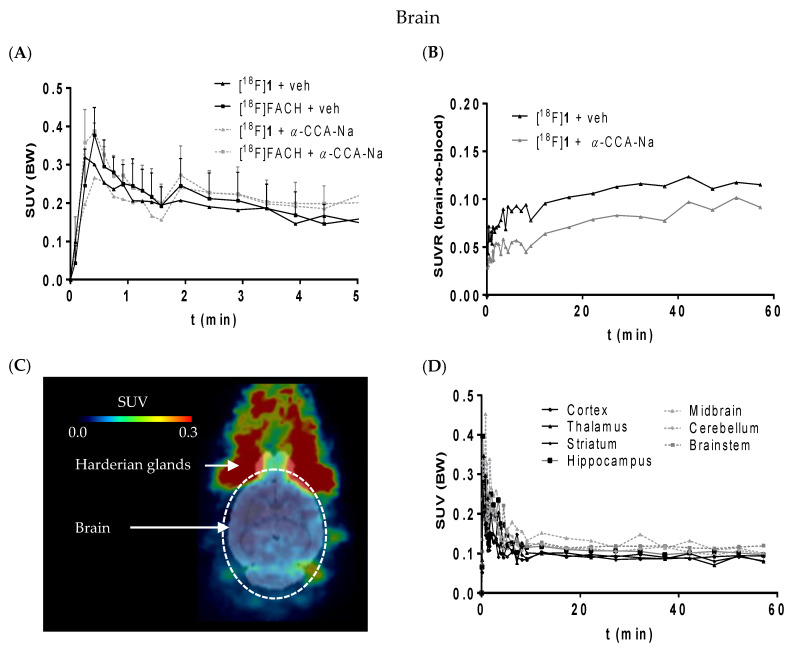
(**A**) Time-activity curve of [^18^F]**1** (n = 1) and [^18^F]FACH (n = 3) representing the SUVs without and with pre-administration of α-CCA-Na; (**B**) The SUVR of [^18^F]**1** showing the brain to blood uptake ratio (n = 1); (**C**) horizontal section of representative PET image (averaged time frames from 0–60 min p.i.) of mouse head; (**D**) SUVs of different brain regions over time after injection of [^18^F]**1**.

According to Figure 7A, [^18^F]**1** shows a similar brain uptake as [^18^F]FACH despite a somewhat higher lipophilicity. Note, however, that this is in accordance with our ACD predictions regarding both log D_7.4_ and log *K*_BB_ values (Table 1). The slightly higher lipophilicity (log *K*_ow_ and log D_7.4_) of **1** is accompanied by a slightly lower BBB penetration (log *K*_BB_), demonstrating further that passive brain uptake is not governed alone by log D_7.4_. Moreover, a selective uptake into a particular brain region could not be verified for both radiotracers (Figure 7, C and D). The SUVR (brain-to-blood) of [^18^F]**1** was reduced by α-CCA-Na by only 25% compared to the control animal (Figure 7B), which is much less than the reduction of kidney uptake and indicates that the uptake of [^18^F]**1** into the brain is partially mediated by MCT1 expressed at the endothelial cells of the BBB [49,50], and partly by non-specific diffusion mediated by lipophilicity. 

Brain capillary endothelial cells are tightly bound to each other and constitute the permeability barrier of the BBB/Neurovascular Unit (NVU), which serves to restrict the transport of compounds into the brain. The permeation of compounds across the BBB/NVU is determined not only by lipophilicity, ionic feature and molecular size, but also by various transporters expressed on the endothelial cell membrane [51]. For example, the radiotracer [^11^C]choline has initially been demonstrated a comparably low brain accumulation with 0.08% of injected dose/g brain (%ID/g) 10 min after injection in mice [52] and 0.15% ID/g at 15 min after injection in rats [53]. This low uptake of [^11^C]choline from blood is mediated by a choline-specific transport system of brain endothelial cell membranes [54]. Despite the low BBB/NVU permeability of [^11^C]choline, it is a clinically relevant radiopharmaceutical for brain tumor imaging with high and specific accumulation in proliferating cancer cells [55,56]. Notably, in our control experiment using the MCT radioligand [^18^F]**1,** a moderate brain accumulation of 0.4% ID/g at 10 min after injection was observed. It has been shown that MCT1 is expressed along with MCT4 in the cerebral microvascular endothelium cells suggesting a key role in transporting endogenous monocarboxylates into and out of the brain [50,57,58]. Together with the important role of MCT1 and MCT4 for glioma metabolism [5,32,33,59], it provides evidence that [^18^F]FACH and/or [^18^F]**1** might be suitable for brain tumor imaging. 

## 3. Materials and Methods

### 3.1. Organic Synthesis

The syntheses of **1**, **2** and **15** (nitro precursor) were implemented by slight modifications of the previously reported procedures [26,36,37,38,39]. All final compounds described here meet the purity requirements determined by HPLC, NMR, and HR-MS analyses.

#### 3.1.1. General Information

Unless otherwise noted, moisture-sensitive reactions were conducted under dry nitrogen or argon. Pd(OAc)_2_, Xantphos (4,5-bis(diphenylphosphino)-9,9-dimethylxanthene), and Cs_2_CO_3_ were purchased from Sigma-Aldrich (SIGMA-ALDRICH Chemie GmbH, Schnelldorf, Germany). Other chemicals and reagents were purchased from commercial sources and were used without further purification. For thin-layer chromatography (TLC), Silica gel 60 F254 plates (Merck KGaA, Darmstadt, Germany) were used. Flash chromatography (fc) was performed using Silica gel 60, 40–64 μm (Merck). Room temperature was 21 °C. ^1^H, ^13^C, and ^19^F spectra were recorded on Varian “MERCURY plus 300/400” (Varian, Palo Alto, CA, USA) and Bruker Advance DRX-400 (Bruker, Billerica, MA, USA); δ in ppm related to tetramethylsilane; coupling constants (*J*) are given with 0.1 Hz resolution. Multiplicities of NMR signals are indicated as follows: s (singlet), d (doublet), t (triplet), q (quartet), m (multiplet), dd (doublet of doublets), ddd (doublet of doublet of doublets), td (triplet of doublets), dq (doublet of quartets) and h (hexet (sextet)). High-resolution mass spectra (HR-MS) were recorded on an FT-ICR APEX II spectrometer (Bruker Daltonics; Bruker Corporation, Billerica, MA, USA) using electrospray ionization (ESI) in positive ion mode.

#### 3.1.2. General Procedures

##### General Procedure A: The Buchwald-Hartwig Aryl Amination Reaction.

Substituted halide (2.5 mmol, 1.0 eq.) was dissolved in dry dioxane (10 mL) in a dry Schlenk tube, under an argon atmosphere. Pd(OAc)_2_ (28 mg, 0.125 mmol, 0.05 eq.), Xantphos (72 mg, 0.125 mmol, 0.05 eq.), and Cs_2_CO_3_ (2.04 g, 6.25 mmol, 2.5 eq.) were added afterwards and the mixture was stirred at 75 °C. After 20 min, amine (3.0 mmol, 1.2 eq.) was added, and the reaction mixture was conducted at 105 °C under an argon atmosphere. The reaction monitored by TLC and it was completed in 30 min. After cooling to room temperature, the reaction mixture was diluted with diethyl ether (Et_2_O), the solids were filtered and washed by Et_2_O. The solvents were evaporated under *vacuum* and the oily residue was then purified by column chromatography.

##### General Procedure B

To a solution of substituted amine (2.0 mmol, 1.0 eq.) in *N,N*-dimethylformamide (DMF, 10 mL), NaH (26.0 mmol of a 60% dispersion in mineral oil, 13.0 eq.) was added in small portions under argon. 1-Iodopropane (0.488 mL, 5.0 mmol, 2.5 eq.) was thereafter added to the mixture. After stirring for 1 h at room temperature, the mixture was slowly poured on an ice-water mixture and stirred for 5 min. The mixture was extracted with ethyl acetate (EtOAc, 2 × 25 mL), the extracts were combined, dried with anhydrous MgSO_4_, and concentrated by evaporation of the solvents under vacuum. The residue was then purified by column chromatography.

##### General Procedure C

POCl_3_ (1.03 mL, 11.0 mmol, 1.1 eq.) was added dropwise to DMF (4.6 mL, 60.0 mmol, 6.0 eq.) at 0 °C and the mixture was stirred 30 min at room temperature. To this solution, *N,N*-disubstituted aniline (10.0 mmol, 1.0 eq.) was added and the reaction mixture was heated up to 80 °C. After 2–4 h, the reaction was quenched by addition of a mixture of ice water, stirred for additional 5 min. The pH was thereafter adjusted to 6–7 by using aqueous 1 M NaOH. The residue was extracted with chloroform (CHCl_3_, 3 × 15 mL), and the organic phases were combined, washed with H_2_O and brine. The extracts were dried with anhydrous MgSO_4_ and the corresponding *N,N*-disubstituted benzaldehyde was purified by column chromatography after evaporation of the solvents. 

To a solution of the substituted benzaldehyde (5.0 mmol, 1.0 eq.) in 20 mL acetonitrile (ACN), cyanoacetic acid (0.991 mL, 15.0 mmol, 3.0 eq.) and piperidine (0.494 mL, 5.0 mmol, 1.0 eq.) were added and refluxed overnight at 85 °C. Upon the completion of the reaction, as judged by TLC, the above solution was poured into a mixture of 3M HCl (10 mL) on ice. The solution was stirred for 30 min and the solids were filtered using a Büchner funnel and washed with ice-cold ACN. The solid was afterwards poured into adequate amount of *n*-hexane and stirred for 15 min to remove the remaining aldehyde. The final compounds were obtained in pure form after filtration of the solids and consequent washing with *n*-hexane.

*6-Fluoro-N-(3-methoxyphenyl)pyridin-2-amine (**5**).* The reaction was carried out according to the general procedure A. Column chromatography: silica, EtOAc/*n*-hexane, 1:2; Yellow oil: 96 % yield; TLC: (silica gel, EtOAc/*n*-hexane, 2:1), R_f_ = 0.8. ^1^H NMR (300 MHz, CDCl_3_) δ 7.56 (q, *J* = 8.0 Hz, 1H), 7.25 (m, 1H), 6.97 (t, *J* = 2.2 Hz, 1H), 6.90 (ddd, *J* = 8.0, 2.1, 0.8 Hz, 1H), 6.73 (dd, *J* = 8.1, 2.3 Hz, 1H), 6.66 (ddd, *J* = 8.3, 2.4, 0.8 Hz, 1H), 6.33 (dd, *J* = 7.8, 2.2 Hz, 1H), 3.83 (s, 3H); ^13^C NMR (75 MHz, CDCl_3_) δ 162.87 (d, *J* = 238.2 Hz), 160.49, 154.92 (d, *J* = 16.1 Hz), 142.12 (d, *J* = 8.4 Hz), 140.82, 130.03, 113.09, 108.87, 106.67, 104.30 (d, *J* = 4.2 Hz),98.29 (d, *J* = 36.1 Hz), 55.27; ^19^F NMR (282 MHz, CDCl_3_) δ −69.08 (d, *J* = 8.1 Hz).

*6-Fluoro-N-(3-methoxyphenyl)-N-propylpyridin-2-amine (**6**)*. The reaction was carried out according to the general procedure B. Column chromatography: silica, EtOAc/*n*-hexane, 1:3; Yellow oil: 95% yield; TLC: (silica gel, EtOAc/*n*-hexane, 1:3), R_f_ = 0.75. ^1^H NMR (400 MHz, CDCl_3_) δ 7.35–7.27 (m, 2H), 6.95–6.65 (m, 3H), 6.12 (td, *J* = 7.6, 2.5 Hz, 2H), 3.85 (d, *J* = 7.6 Hz, 2H), 3.81 (s, 3H), 1.66 (m, 2H), 0.92 (t, *J* = 7.4 Hz, 3H); ^13^C NMR (101 MHz, CDCl_3_) δ 162.85 (d, *J* = 234.7 Hz), 160.87, 157.87 (d, *J* = 16.1 Hz), 145.82, 140.72 (d, *J* = 8.3 Hz), 130.50, 120.09, 113.64, 111.95, 104.78 (d, *J* = 4.1 Hz), 95.17 (d, *J* = 37.4 Hz), 55.30, 51.76, 21.05, 11.26; ^19^F NMR (377 MHz, CDCl_3_) δ −69.25 (d, *J* = 8.3 Hz).

*6-Fluoro-N-(3-methoxyphenyl)-N-phenylpyridin-2-amine (**7**).* The reaction was carried out according to the general procedure A. For this compound, higher amounts of Pd(OAc)_2_ (84 mg, 0.375 mmol, 0.15 eq.) and Xantphos (217 mg, 0.375 mmol, 0.15 eq.) were added stepwise (3 × 0.125 mmol) to the reaction mixture over 24 h. Column chromatography: silica, EtOAc/n-hexane, 1:3; Milky oil: 46% yield; TLC: (silica gel, EtOAc/n-hexane, 1:3), R_f_ = 0.85. ^1^H NMR (400 MHz, CDCl_3_) δ 7.48 (dt, *J* = 8.4, 7.9 Hz, 1H), 7.38–7.29 (m, 2H), 7.28–7.12 (m, 4H), 6.79 (ddd, *J* = 7.9, 2.0, 0.9 Hz, 1H), 6.77–6.69 (m, 2H), 6.50 (ddd, *J* = 8.1, 2.2, 0.5 Hz, 1H), 6.34 (ddd, *J* = 7.8, 2.9, 0.5 Hz, 1H), 3.75 (s, 3H); ^13^C NMR (101 MHz, CDCl_3_) δ 162.43 (d, *J* = 237.8 Hz), 160.49, 157.50 (d, *J* = 15.1 Hz), 146.32, 145.06, 141.55 (d, *J* = 8.1 Hz), 130.02, 129.39, 126.62, 125.23, 119.07, 112.63, 110.74, 109.10 (d, *J* = 4.5 Hz), 99.41 (d, *J* = 37.0 Hz), 55.29; ^19^F NMR (377 MHz, CDCl_3_) δ −67.46 (d, *J* = 7.3 Hz).

*4-((6-Fluoropyridin-2-yl)(propyl)amino)-2-methoxybenzaldehyde (**8**).* The reaction was carried out according to the general procedure C. Column chromatography: silica, EtOAc/petroleum ether (PE), 1:2; Yellow oil: 57% yield; TLC: (silica gel, EtOAc/PE, 1:2), R_f_ = 0.55. ^1^H NMR (300 MHz, CDCl_3_) δ 10.35 (s, 1H), 7.82 (d, *J* = 8.4 Hz, 1H), 7.46 (dt, *J* = 8.5, 7.9 Hz, 1H), 6.88 (ddd, *J* = 8.4, 2.0, 0.7 Hz, 1H), 6.82 (d, *J* = 1.9 Hz, 1H), 6.55 (ddd, *J* = 8.1, 2.4, 0.5 Hz, 1H), 6.30 (ddd, *J* = 7.8, 2.9, 0.5 Hz, 1H), 3.98–3.89 (m, 2H), 3.88 (s, 3H), 1.69 (dq, *J* = 14.8, 7.4 Hz, 2H), 0.94 (t, *J* = 7.4 Hz, 3H); ^13^C NMR (75 MHz, CDCl_3_) δ 189.19, 163.05 (d, *J* = 244.3 Hz), 161.72, 159.68 (d, *J* = 15.1 Hz), 149.95, 140.12 (d, *J* = 8.1 Hz), 130.33, 118.77, 111.37 (d, *J* = 4.5 Hz), 106.62, 100.47 (d, *J* = 37.0 Hz), 101.76, 55.99, 49.89, 20.95, 11.38; ^19^F NMR (282 MHz, CDCl_3_) δ −68.09 (d, *J* = 8.6 Hz).

*4-((6-Fluoropyridin-2-yl)(phenyl)amino)-2-methoxybenzaldehyde (**9**)*. The reaction was carried out according to the general procedure C. Column chromatography: silica, EtOAc/*n*-hexane, 1:3; Yellow oil: 68% yield; TLC: (silica gel, EtOAc/*n*-hexane, 1:3), R_f_ = 0.60. ^1^H NMR (300 MHz, CDCl_3_) δ 10.32 (s, 1H), 7.73 (d, *J* = 8.5 Hz, 1H), 7.59 (q, *J* = 8.0 Hz, 1H), 7.46–7.37 (m, 2H), 7.33–7.25 (m, 1H), 7.24–7.16 (m, 2H), 6.78 (d, *J* = 2.0 Hz, 1H), 6.69 (ddd, *J* = 8.5, 2.0, 0.8 Hz, 1H), 6.60 (dd, *J* = 8.0, 1.7 Hz, 1H), 6.54–6.46 (m, 1H), 3.77 (s, 3H); ^13^C NMR (75 MHz, CDCl_3_) δ 188.37, 162.91 (d, *J* = 235.1 Hz), 162.63,156.61 (d, *J* = 14.9 Hz), 152.14, 144.37, 142.13 (d, *J* = 8.1 Hz), 130.06, 129.50, 127.66, 126.70, 120.65, 116.19, 111.59 (d, *J* = 4.6 Hz), 106.62, 101.86 (d, *J* = 36.6 Hz), 55.60; ^19^F NMR (282 MHz, CDCl_3_) δ −66.92 (d, *J* = 8.5 Hz).

*(E)-2-Cyano-3-(4-((6-fluoropyridin-2-yl)(propyl)amino)-2-methoxyphenyl)acrylic Acid (**1**).* The reaction was carried out according to the general procedure C. Yellow solid: 95% yield; TLC: (silica gel, CHCl_3_/MeOH/acetic acid (AcOH), 95:5:0.1), R_f_ = 0.60. ^1^H NMR (400 MHz, CDCl_3_/CD_3_OD) δ 8.63 (s, 1H), 8.30 (d, *J* = 8.7 Hz, 1H), 7.46 (q, *J* = 8.2 Hz, 1H), 6.86 (dd, *J* = 8.7, 2.1 Hz, 1H), 6.74 (d, *J* = 2.1 Hz, 1H), 6.59 (dd, *J* = 8.1, 2.2 Hz, 1H), 6.31 (dd, *J* = 7.8, 2.8 Hz, 1H), 3.93–3.86 (m, 2H), 3.80 (s, 3H), 1.65 (h, *J* = 7.4 Hz, 2H), 0.90 (t, *J* = 7.4 Hz, 3H); ^13^C NMR (101 MHz, CDCl_3_/CD_3_OD) δ 164.86, 162.72 (d, *J* = 237.5 Hz), 160.52, 156.27 (d, *J* = 15.2 Hz), 151.55, 148.32, 141.46 (d, *J* = 8.3 Hz), 133.13, 130.43, 116.63, 116.39, 108.28 (d, *J* = 4.3 Hz), 106.26, 100.01, 99.02 (d, *J* = 36.9 Hz), 55.72, 51.83, 21.14, 11.14; ^19^F NMR (376 MHz, CDCl_3_/CD_3_OD) δ -68.23 (dt, *J* = 8.3, 2.2 Hz).HR-MS (ESI) *m*/*z*:calcd. for [C_18_H_17_FN_3_O]^+^ = 310.1350; found = 310.1332 [M–CO_2_H]^+^.

*(E)-2-Cyano-3-(4-((6-fluoropyridin-2-yl)(phenyl)amino)-2-methoxyphenyl)acrylic Acid (**2**).* The reaction was carried out according to the general procedure C. Orange solid: 98% yield; TLC: (silica gel, CHCl_3_/MeOH/AcOH), 95:5:0.1), R_f_ = 0.75.^1^H NMR (400 MHz, CDCl_3_) δ 8.75 (s, 1H), 8.37 (d, *J* = 8.8 Hz, 1H), 7.65 (q, *J* = 8.0 Hz, 1H), 7.47 (t, *J* = 7.7 Hz, 2H), 7.41–7.17 (m, 3H), 6.80 (d, *J* = 1.9 Hz, 1H), 6.73 (dd, *J* = 8.8, 1.9 Hz, 1H), 6.69–6.61 (m, 1H), 6.58 (dd, *J* = 7.9, 2.7 Hz, 1H), 3.79 (s, 3H); ^13^C NMR (101 MHz, CDCl_3_) δ 167.91, 162.31 (d, *J* = 240.5 Hz), 160.76, 156.25 (d, *J* = 14.6 Hz), 152.17, 149.60, 143.99, 142.34 (d, *J* = 8.2 Hz), 130.35, 130.24, 127.87, 127.16, 116.26, 115.94, 115.58, 112.29 (d, *J* = 4.5 Hz), 105.47, 102.54 (d, *J* = 36.5 Hz), 97.31, 55.82; ^19^F NMR (377 MHz, CDCl_3_) δ -66.88 (d, *J* = 8.2 Hz).HR-MS (ESI) m/z: calcd. for [C_21_H_15_FN_3_O]^+.^ = 344.1194; found = 344.1180 [M–CO_2_H]^+^.

*N-(3-Methoxyphenyl)-6-nitropyridin-2-amine (**12**).* The reaction was carried out according to the general procedure A. Column chromatography: silica, EtOAc/*n*-hexane, 1:1; Yellow solid: 80 % yield; TLC: (silica gel, EtOAc/*n*-hexane, 1:1), R_f_ = 0.65. ^1^H NMR (400 MHz, CDCl_3_) δ 7.78–7.70 (m, 1H), 7.63 (dd, *J* = 7.6, 0.6 Hz, 1H), 7.31–7.24 (m, 1H), 7.23–7.19 (m, 1H), 7.14 (dd, *J* = 8.2, 0.6 Hz, 1H), 6.88 (ddd, *J* = 7.9, 2.0, 0.8 Hz, 1H), 6.71 (ddd, *J* = 8.3, 2.5, 0.8 Hz, 1H), 3.86 (s, 3H); ^13^C NMR (101 MHz, CDCl_3_) δ 160.29, 155.31, 155.06, 140.46, 139.94, 129.79, 114.77, 112.21, 109.24, 107.35, 105.65, 55.16.

*N-(3-Methoxyphenyl)-6-nitro-N-propylpyridin-2-amine (**13**).* The reaction was carried out according to the general procedure B. Column chromatography: silica, EtOAc/*n*-hexane, 1:3; Yellow oil: 93% yield; TLC: (silica gel, EtOAc/*n*-hexane, 1:3), R_f_ = 0.70. ^1^H NMR (400 MHz, CDCl_3_) δ 7.53–7.29 (m, 3H), 6.88 (dd, *J* = 8.3, 2.3 Hz, 1H), 6.82 (d, *J* = 7.9 Hz, 1H), 6.79–6.68 (m, 1H), 6.59 (d, *J* = 8.2 Hz, 1H), 3.95 (t, *J* = 7.4 Hz, 2H), 3.82 (s, 3H), 1.69 (h, *J* = 7.4 Hz, 2H), 0.95 (t, *J* = 7.4 Hz, 3H); ^13^C NMR (101 MHz, CDCl_3_) δ 161.44, 157.92, 156.25, 145.36, 139.22, 131.23, 120.24, 114.28, 113.93, 112.86, 105.78, 55.72, 52.38, 21.21, 11.68.

*2-Methoxy-4-((6-nitropyridin-2-yl)(propyl)amino)benzaldehyde (**14**)*. The reaction was carried out according to the general procedure C. Column chromatography: silica, EtOAc/PE, 1:2; Yellow oil: 92% yield; TLC: (silica gel, EtOAc/PE, 1:2), R_f_ = 0.60. ^1^H NMR (400 MHz, CDCl_3_) δ 10.40 (s, 1H), 7.89 (d, *J* = 8.3 Hz, 1H), 7.63–7.53 (m, 2H), 6.94–6.89 (m, 2H), 6.88 (d, *J* = 1.9 Hz, 1H), 4.03 (t, *J* = 7.4 Hz, 2H), 3.91 (s, 3H), 1.74 (h, *J* = 7.4 Hz, 2H), 0.97 (t, *J* = 7.4 Hz, 3H); ^13^C NMR (101 MHz, CDCl_3_) δ 188.37, 163.12, 156.66, 155.98, 151.33, 139.56, 130.32, 122.69, 118.05, 115.38, 108.86, 107.32, 55.84, 52.30, 21.08, 11.38.

*(E)-2-Cyano-3-(2-methoxy-4-((6-nitropyridin-2-yl)(propyl)amino)phenyl)acrylic Acid (**15**).* The reaction was carried out according to the general procedure C. Yellow solid: >98% yield; TLC: (silica gel, CHCl_3_/MeOH/AcOH), 95:5:0.1), R_f_ = 0.70. ^1^H NMR (400 MHz, CDCl_3_/CD_3_OD) δ 8.62 (s, 1H), 8.30 (d, *J* = 8.5 Hz, 1H),7.58 (t, *J* = 7.9 Hz, 1H), 7.51 (d, *J* = 7.6 Hz, 1H), 6.92 (d, *J* = 8.3 Hz, 1H), 6.89 (dd, *J* = 8.6, 1.7 Hz, 1H), 6.80 (d, *J* = 1.5 Hz, 1H), 3.98 (t, *J* = 7.4 Hz, 2H), 3.82 (s, 3H), 1.68 (h, *J* = 7.4 Hz, 2H), 0.91 (t, *J* = 7.3 Hz, 3H); ^13^C NMR (101 MHz, CDCl_3_/CD_3_OD) δ 164.39, 160.51, 156.43, 155.70, 150.30, 148.24, 139.62, 130.62, 118.14, 117.66, 116.35, 115.78, 107.90, 107.54, 101.63, 55.82, 52.13, 20.95, 11.16. HR-MS (ESI) *m*/*z* calcd. for [C_18_H_17_N_4_O_3_]^+**.**^= 337.1295; found = 337.1283 [M–CO_2_H]^+**.**^.

### 3.2. Assessment of the MCT1 Inhibition by [^14^C]lactate Uptake Assay 

Inhibition of the MCT1-mediated lactate transport was determined using the rat brain endothelial cell line (RBE4, a gift from F. Roux research group [40]) as previously described [26]. PCR analysis and Western Blot demonstrated that only the MCT1 isoform is expressed by RBE4 cells.

### 3.3. Radiochemistry

#### 3.3.1. General Methods

No-carrier-added [^18^F]fluoride was produced via the [^18^O(p,n)^18^F] nuclear reaction by irradiation of an [^18^O]H_2_O target (Hyox 18 enriched water, Rotem Industries Ltd., Arava, Israel) on a Cyclone 18/9 (IBA RadioPharma Solutions, Lourain-La-Neuve, Belgium) with fixed energy proton beam using Nirta [^18^F]fluoride XL target. Radio thin layer chromatography (radio-TLC) was performed on silica gel (ALUGRAM SIL G/UV254, Machery-Nagel, Düren, Germany) pre-coated plates with a mixture of dichloromethane (DCM)/MeOH (4:1) as eluent. The plates were exposed to storage phosphor screens (BAS IP MS 2025 E, GE Healthcare Europe GmbH, Freiburg, Germany) and recorded using the Amersham Typhoon RGB Biomolecular Imager (GE Healthcare Life Sciences). Images were quantified with the ImageQuant TL8.1 software (GE Healthcare Life Sciences). 

Analytical chromatographic separations were performed on a JASCO LC-2000 system, incorporating a PU-2080Plus pump, AS-2055Plus auto injector (100 µL sample loop), and a UV-2070Plus detector coupled with a gamma radioactivity HPLC flow detector (Gabi Star, raytest Isotopenmessgeräte GmbH, Straubenhardt, Germany). Data analysis was performed with the Galaxy chromatography software (version 1.10.0.5590, Agilent Technologies Deutschland GmbH, Waldbronn, Germany) using the chromatograms obtained at 254 and 370 nm. Reprosil-Pur C18-AQ column (250 × 4.6 mm; 5 µm; Dr. Maisch HPLC GmbH; Ammerbuch-Entingen, Germany) with an eluent mixture of ACN/20 mM NH_4_OAc (aq., pH 6.8) and a flow of 1.0 mL/min were used (gradient: eluent A 10% ACN/20 mM NH_4_OAc (aq.); eluent B 90% ACN/20 mM NH_4_OAc (aq.); 0–5 min 100% A, 5–20 min up to 62% B, 20–21 min up to 100% B, 21–26 min 100% B, 26–27 min up to 100% A, 27–35 min 100% A; isocratic: 30% MeOH/20 mM NH_4_OAc (aq.), flow: 0.75 mL/min, UV detection at 370 nm).

Semi-preparative HPLC separation was performed by using the HPLC system implemented in the TRACERlab FX2 N synthesizer (GE Healthcare). A Reprosil-Pur 120 CN column (250 × 20 mm, 10 µm, Dr. Maisch HPLC GmbH, Germany) was used with 45% MeOH/20 mM NH_4_OAc (aq.) as eluent mixture and a flow rate of 7.0 mL/min. The ammonium acetate concentration stated as aq. 20 mM NH_4_OAc, corresponds to the concentration in the aqueous component of an eluent mixture. 

The molar activities were determined based on a calibration curve carried out under isocratic HPLC conditions (Reprosil-Pur C18-AQ, 250 × 4.6 mm, 30% MeOH/20 mM NH_4_OAc (aq.), flow: 0.75 mL/min) using chromatograms obtained at 370 nm as an appropriate maximum of UV absorbance.

#### 3.3.2. Radiosynthesis of [^18^F]**1**

No carrier added [^18^F]fluoride in 1.5 mL water was trapped on a Sep-Pak Accell Plus QMA Carbonate Plus light cartridge (Waters GmbH, Eschborn, Germany). The activity was eluted with 400 µL of an aqueous solution of potassium carbonate (K_2_CO_3_, 1.8 mg, 13 µmol) into a 4 mL V-shape vial prefilled with Kryptofix^®^ 2.2.2 (K_2.2.2_, 11 mg, 29 µmol) in 1 mL ACN. The aqueous [^18^F]fluoride was azeotropically dried under vacuum and nitrogen flow within 7–10 min using a single mode microwave (75 W, at 50–60 °C, power cycling mode; Discover PETWave from CEM GmbH, Kamp-Lintfort, Germany) [60]. Two aliquots of ACN (2 × 1.0 mL) were added during the drying procedure and the final complex was obtained in a dried form. A solution of 1.0 mg of the nitro precursor **15** in 750 µL DMSO was added, and the ^18^F-labeling was performed at 130 °C. To determine the radiochemical yields of the labeling process, samples were taken at different time points (5, 10, 15, and 20 min) and analyzed by radio-HPLC and radio-TLC. 

After cooling to < 30 °C, the reaction mixture was diluted with 2.0 mL aqueous NH_4_HCO_2_ (adjusted to pH 4 with formic acid) and 2.0 mL MeOH/water (1:/1) and directly applied to an isocratic semi-preparative RP-HPLC for isolation of [^18^F]**1**. The collected radiotracer fraction was diluted with 40 mL water to perform final purification by sorption on a Sep-Pak^®^ C18 light cartridge (Waters, GmbH, Eschborn, Germany) and successive elution with 1.3 mL of ethanol. The ethanolic solution was concentrated under a gentle argon stream at 70 °C to a final volume of 10–50 µL. Afterwards, the radiotracer was diluted in isotonic saline to obtain a final product containing 10% of EtOH (*v*/*v*).

#### 3.3.3. Determination of In Vitro Stability and log D_7.4_ of [^18^F]**1**

In vitro stability was investigated by incubation of about 5 MBq of [^18^F]**1** in phosphate-buffered saline (PBS, pH 7.4), *n*-octanol, saline and ethanol (500 µL) at 40 °C up to 60 min. Samples were taken at 60 min and the radio-TLC and radio-HPLC analyses were performed.

The lipophilicity of [^18^F]**1** was experimentally determined by partitioning a small amount of the radiotracer between *n*-octanol and PBS (pH 7.4) at room temperature using the conventional shake-flask method. The radiotracer (15 µL, ~800 kBq) was added to the tubes containing a mixture of *n*-octanol and PBS (3.0 mL, n = 4). After shaking for 20 min using the mechanical shaker (HS250 basic, IKA Labortechnik GmbH & Co. KG, Staufen, Germany), the samples were centrifuged for 5 min at 5000 rpm, followed by separation of the phases. Aliquots were taken from the organic and the aqueous phases (1 mL of each) and activity was measured in a γ-counter (PerkinElmer Wallac Wizard 1480 Gamma Counter, manufactured by WALLAC, Turku, Finland). For the second extraction, another 1 mL aliquot of the organic layer was mixed with 2.0 mL *n*-octanol and 3.0 mL of PBS and was subjected to the same procedure. The distribution coefficient (D) was calculated as [activity (cpm/mL) in *n*-octanol]/[activity (cpm/mL) in PBS, pH 7.4] stated as the decade logarithm (log D_7.4_).

### 3.4. Animal Studies

#### 3.4.1. General Information

All experimental work including animals has been conducted in accordance with the national legislation on the use of animals for research (Tierschutzgesetz (TierSchG), Tierschutz-Versuchstierverordnung (TierSchVersV)) and has been approved by the responsible research ethics committee (TVV 18/18, DD24.1-5131/446/19, Landesdirektion Sachsen, 20 June, 2018). Female CD-1 mice, 10–12 weeks, were obtained from the Medizinisch-ExperimentellesZentrum at Universität Leipzig. For the time of the experiments, the animals were kept in a dedicated climatic chamber with free access to water and food under a 12:12 h dark:light cycle at a constant temperature of 24 °C.

#### 3.4.2. In Vitro Autoradiographic Analysis of Binding Sites of [^18^F]**1** in Mouse Kidney

The kidneys was isolated after cervical dislocation from one CD-1 mouse, frozen rapidly in isopentane at −25 °C for 5 min, and stored at −25 °C until the sectioning. Cryosections (transversal, 10 µm) were obtained using a microtome (MICROM HM560, Thermo Scientific Microm, Fisher Scientific GmbH, Schwerte, Germany), mounted on microscopy slides (SuperFrost, Thermo Scientific Menzel, Fisher Scientific GmbH, Schwerte, Germany), dried for ~2 h at room temperature, and stored at –25 °C until the autoradiography study. For the experiment, the slides were taken out from the freezer, the cryosections dried under a stream of cold air, and pre-incubated with PBS for 15 min at room temperature. The incubation solution was decanted, the slices dried again under a stream of cold air, and covered afterwards with the incubation solution ([^18^F]**1**, 198 kBq/mL PBS or 1.19 nM at the time of incubation, without (total binding) or with co-incubation with 10^−5^, 10^−7^, and 10^−9^ M α-CHC-Na). Incubation at room temperature was terminated after 60 min, the slides were washed twice in 50 mM TRIS-HCl, pH 7.4 at 4 °C, on ice for two minutes each followed by dipping in ice-cold demineralized water for 5 s and rapid drying under a stream of cold air. Afterwards, the slides were exposed to a phosphor imager plate (BAS-IP TR 2025, FujiFilm Corporation, Tokyo, Japan) along with standards obtained by pipetting and drying 1 µL of each concentration of a serial dilution of the radiotracer solution on to a microscopic slide.

#### 3.4.3. Analysis of Radiometabolites of [^18^F]**1** in Mouse

The radiotracer [^18^F]**1** (32.84 MBq in 200 μL isotonic saline) was injected in an awake female CD-1 mouse (12 weeks old, 31 g) via the tail vein. After 30 min, the animal was slightly anesthetized with isoflurane, and blood was collected from retro-orbital bleeding followed by cervical dislocation. Blood plasma was separated by centrifugation of the blood sample at 10,000 rpm for 2 min. Brain was also isolated, cleaned by pouring PBS and homogenized in demineralized water (~2 mL/g tissue) in a borosilicate glass with a PTFE plunger by 10 strokes at 1000 rpm (POTTER S, Homogenizer, B. Braun Biotech, Sartorius AG, Göttingen, Germany). Blood plasma and brain homogenate were provided for preparation of samples for radio-HPLC analyses as described in the following.

RP-HPLC: Protein precipitation was performed by addition of an ice-cold ACN/H_2_O mixture (9:1) to the plasma and brain samples in a ratio of 4:1 (*v*/*v*: solvent/tissue sample, n = 2). The samples were vortexed for 2 min, incubated on ice for 3 min, and the suspensions were centrifuged at 10,000 rpm at 4 °C for 5 min. For the second extraction, the precipitates were re-dissolved in 100 μL of the solvent mixture, vortexed for 3 min, incubated on ice for 5 min and subjected to the same centrifuging procedure. The combined supernatants (total volume between 1–2 mL) were concentrated at 70 °C under argon flow to a final volume of approximately 100 μL and were analyzed by analytical radio-HPLC. A Reprosil-Pur C18-AQ column (250 mm × 4.6 mm; 5 μm) was used as stationary phase and elution was done using the gradient mode as described in Section 3.3.1. To determine the percentage of radioactivity in the supernatants compared to total activity, aliquots of each step as well as the precipitates were quantified by gamma counting.

MLC: Preparation of the plasma and brain samples was performed as already described [47,48]. A Reprosil-Pur C18-AQ column (250 × 4.6 mm, particle size: 10 µm) coupled with a pre-column of 10 mm length was used. Separations were performed by using an isocratic mode with an aqueous eluent containing 50 mM sodium dodecyl sulfate and 10 mM Na_2_HPO_4_ at a flow rate of 1.0 mL/min. 

#### 3.4.4. Small Animal Micro-PET/MR Studies

The dynamic biodistribution of the radiotracers was assessed by small animal PET (nanoscan, Mediso, Hungary) over 60 min PET with a subsequently T1 weighted MR. Anaesthetized (2% isofluran, carrier gas mixture of 40% air and 60% O_2_) female CD-1 mice (bodyweight = 30.3 ± 1.1 g) were kept during imaging on a heated animal bed to sustain body temperature and were pretreated with vehicle (0.9% saline) and α-CHC-Na (25 mg/kg bodyweight) prior to tracer application ([^18^F]**1**: 5.8 ± 0.2 MBq, 1.1 fmol/g bodyweight and [^18^F]FACH: 5.9 ± 0.5 MBq, 3.7 ± 1.8 fmol/g), whereby all injections were administered intravenously. The acquisitions were performed in normal mode and a coincidence Mode 1–5. For subsequent dynamic reconstructions, list mode data were sorted into sinograms (12 × 10 s, 6 × 30 s, 5 × 60 s, and 10 × 300 s). The frames were reconstructed by Ordered Subset Expectation Maximization applied to 3D sinograms (OSEM3D) with an attenuation correction with 4 iterations, 6 subsets and a voxel size of 0.4 mm^3^ (Nucline v2.01, Mediso, Hungary). Analyses of reconstructed studies were performed with PMOD software (v4.005, PMOD Technologies LLC, Zurich, Switzerland) and results are expressed in Standardized Uptake Value (SUV). 

## 4. Conclusions

In summary, two new analogs of FACH, **1** and **2**, were synthesized and the former, with moderate MCT1 inhibition, was regarded to be a good PET candidate and therefore chosen for labeling with fluorine-18. Although the partition coefficient log D_7.4_ of [^18^F]**1** was 2-fold higher than the one of [^18^F]FACH, the brain accumulation of both radiotracers was in a similar moderate range. This demonstrates that log D_7.4_ alone does not govern passive diffusion into the brain, which is also reflected by comparing log D_7.4_ with calculated log *K*_BB_ (brain-blood partition coefficient). Nevertheless, the high uptake of [^18^F]**1** in kidney and other peripheral MCT-expressing organs together with the strong inhibition by specific drugs provide evidence that this radiotracer is suitable for future investigation of MCT imaging with PET. Moreover, these results suggest that further structural modifications towards improving MCT-mediated transport might result in higher brain uptake in vivo. 

## 5. Patents

The nitro- or bromo- precursor for the radiosynthesis of the structurally modified analogs of FACH is subject of a patent application by Helmholtz-Zentrum Dresden-Rossendorf (DMPA registration No. 10 2019 112 040.3) with following contributing inventors: Rareş-Petru Moldovan, Masoud Sadeghzadeh, Barbara Wenzel, Mathias Kranz, Steffen Fischer, Rodrigo Teodoro, Friedrich-Alexander Ludwig, Magali Toussaint and Peter Brust. 

## Figures and Tables

**Table 1:** Calculated physicochemical parameters of our drug candidates and structurally related compounds.^1^

Compound	log *K*_ow_	log D_7.4_	*f*_u_	p*K*_a_	log *K*_BB_
FACH	4.43	0.69	4.5 × 10^−8^	0.05	−0.10
Compound A	5.41	1.66	2.5 × 10^−7^	0.80	−0.57
**1**	4.83	1.08	7.4 × 10^−8^	0.27	−0.49
**2**	5.07	1.32	2.1 × 10^−7^	0.73	−1.05
Cinnamic acid	2.07	−0.91	8.7 × 10^−4^	4.34	−0.14
α-CN cinnamic acid	2.27	−1.48	1.6 × 10^−7^	0.60	−0.54
α-CCA	1.79	−1.96	2.8 × 10^−7^	0.85	−0.45

^1^ The decadic logarithms of the *n*-octanol/water partition coefficient (log *K*_ow_) have been calculated with EPI Suite [42], and these values have been employed for predicting the p*K*_a_-pH-corrected *n*-octanol/water distribution coefficients, log D_7.4_. For the latter, the ACD approach log *K*_ow_ (ionized) = log *K*_ow_ (unionized) − 3.75 (applicable for the relevant range of log *K*_ow_ data) [43,44] with D_7.4_ = *f*_u_ × *K*_ow_ + (1 − *f*_u_) × *K*_ow_ (ionized) has been employed (see also [44]). Note that the ACD-calculated log *K*_ow_ data are lower by 0.5–1.5 log units except for a slightly larger value for cinnamic acid, resulting in correspondingly lower calculated log D_7.4_ data. Moreover, *f*_u_ denotes the compound fraction unionized at pH 7.4 according to the Henderson-Hasselbalch relationship, and p*K*_a_ as well as the brain-blood partition coefficient (*K*_BB_) have been calculated with the ACD software.

## Supplementary Materials

The following are available online. Figure S1: (**A**) Representative semi-preparative radio- and UV-HPLC chromatograms representing two peaks a/b which are supposed to reflect neutral and deprotonated form of the radiotracer ([^18^F]**1a/b**) (conditions: Reprosil-Pur 120 CN, 10 µm, 250 × 20 mm, 45% MeOH/20 mM NH_4_OAc (aq.), 7.0 mL/min); (**B**) Analytical radio- and UV-HPLC chromatograms detected two peaks a/b which assumed to be the neutral and deprotonated form of the final product ([^18^F]**1a/b**) in the sample solution co-eluted with the non-radioactive reference 1 (conditions: Reprosil-Pur C18-AQ, 250 × 4.6 mm, gradient with an eluent mixture of ACN/20 mM NH_4_OAc (aq.), 1.0 mL/min)., Figure S2: Analytical UV- and radio-HPLC chromatograms representing two peaks a/b which are supposed to reflect the neutral and deprotonated form of the radiotracer ([^18^F]**1a/b**) in the mouse brain sample at 30 min p.i. measured under reversed-phase conditions (Reprosil-Pur C18-AQ, 250 × 4.6 mm, gradient with an eluent mixture of ACN/20 mM NH_4_OAc (aq.), 370 nm, 1.0 mL/min).
