# Supplementary material for: Development of Novel Analogs of the Monocarboxylate Transporter Ligand FACH and Biological Validation of One Potential Radiotracer for Positron Emission Tomography (PET) Imaging
Source: Molecules. 2020 May 14;25(10):2309. doi: 10.3390/molecules25102309 (PMC7288138; doi:10.3390/molecules25102309)
Supplement: Supplementary file 1 [file molecules-25-02309-s001.pdf]

Supplementary Materials: Development of Novel Analogs of the Monocarboxylate Transporter Ligand FACH and Biological Validation of One Potential Radiotracer for Positron Emission Tomography (PET) Imaging

Masoud Sadeghzadeh, Barbara Wenzel, Daniel Gündel, Winnie Deuther-Conrad, Magali Toussaint, Rareş-Petru Moldovan, Steffen Fischer, Friedrich-Alexander Ludwig, Rodrigo Teodoro, Shirisha Jonnalagadda, Sravan K. Jonnalagadda, Gerrit Schüürmann, Venkatram R. Mereddy, Lester R. Drewes and Peter Brust

Affiliations and contributions of the authors are listed in the main manuscript.

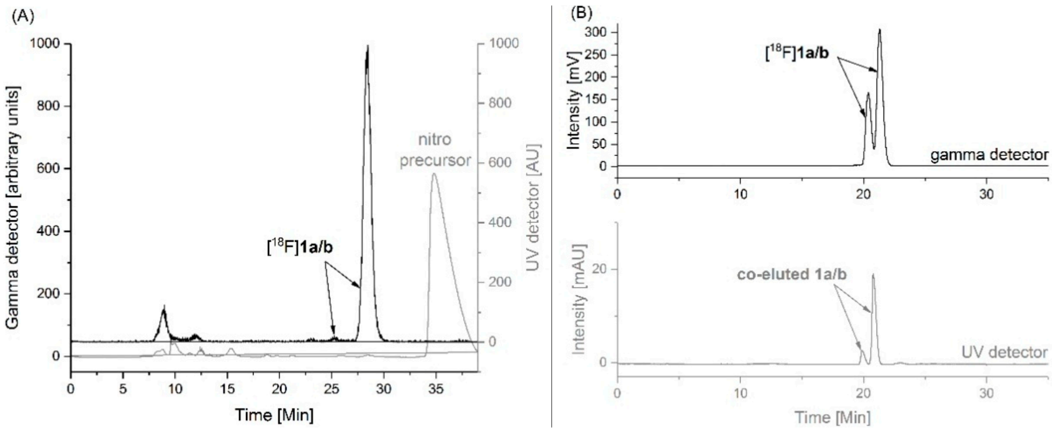

Figure S1. (**A**) Representative semi-preparative radio- and UV-HPLC chromatograms representing two peaks a/b which are supposed to reflect neutral and deprotonated form of the radiotracer ([^{18}F]**1a/b)** (conditions: Reprosil-Pur 120 CN, 10 μm, 250 × 20 mm, 45% MeOH/20 mM NH_4OAc (aq.), 7.0 mL/min); (**B**) Analytical radio- and UV-HPLC chromatograms detected two peaks a/b which assumed to be the neutral and deprotonated form of the final product ([^{18}F]**1a/b**) in the sample solution co-eluted with the non-radioactive reference **1** (conditions: Reprosil-Pur C18-AQ, 250 × 4.6 mm, gradient with an eluent mixture of ACN/20 mM NH_4OAc (aq.), 1.0 mL/min).

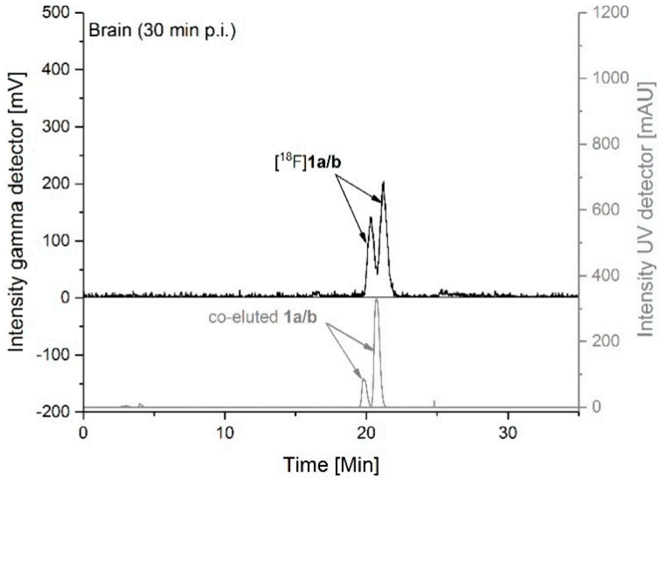

Figure S2. Analytical UV- and radio-HPLC chromatograms representing two peaks a/b which are supposed to reflect the neutral and deprotonated form of the radiotracer ([^{18}F]**1a/b**) in the mouse brain sample at 30 min p.i. measured under reversed-phase conditions (Reprosil-Pur C18-AQ, 250 × 4.6 mm, gradient with an eluent mixture of ACN/20 mM NH_4OAc (aq.), 370 nm, 1.0 mL/min).
